# A Systematic Role of Metabolomics, Metabolic Pathways, and Chemical Metabolism in Lung Cancer

**DOI:** 10.3390/vaccines11020381

**Published:** 2023-02-07

**Authors:** Sandra Kannampuzha, Anirban Goutam Mukherjee, Uddesh Ramesh Wanjari, Abilash Valsala Gopalakrishnan, Reshma Murali, Arunraj Namachivayam, Kaviyarasi Renu, Abhijit Dey, Balachandar Vellingiri, Harishkumar Madhyastha, Raja Ganesan

**Affiliations:** 1 Department of Biomedical Sciences, School of Biosciences and Technology, Vellore Institute of Technology (VIT), Vellore 632014, India; sandrajacobkannampuzha@gmail.com (S.K.); mukherjee1anirban@gmail.com (A.G.M.); uddeshwanjari786@gmail.com (U.R.W.); reshmamurali.rm@gmail.com (R.M.); arunrajnamashivayam97@gmail.com (A.N.); 2 Centre of Molecular Medicine and Diagnostics (COMManD), Department of Biochemistry, Saveetha Dental College & Hospitals, Saveetha Institute of Medical and Technical Sciences, Saveetha University, Chennai 600077, India; kaviyarasirenu.92@gmail.com; 3 Department of Life Sciences, Presidency University, Kolkata 700073, India; abhijit.dbs@presiuniv.ac.in; 4 Stem Cell and Regenerative Medicine/Translational Research, Department of Zoology, School of Basic Sciences, Central University of Punjab (CUPB), Bathinda 151401, India; balachandar.vellingiri@cup.edu.in; 5 Department of Cardiovascular Physiology, Faculty of Medicine, University of Miyazaki, Miyazaki 889-1692, Japan; hkumar@med.miyazaki-u.ac.jp; 6 Institute for Liver and Digestive Diseases, College of Medicine, Hallym University, Chuncheon 24252, Republic of Korea

**Keywords:** lung cancer, metabolites, pathways, Warburg effect, glycolysis, amino acids, lipids

## Abstract

Lung cancer (LC) is considered as one of the leading causes of cancer-associated mortalities. Cancer cells’ reprogrammed metabolism results in changes in metabolite concentrations, which can be utilized to identify a distinct metabolic pattern or fingerprint for cancer detection or diagnosis. By detecting different metabolic variations in the expression levels of LC patients, this will help and enhance early diagnosis methods as well as new treatment strategies. The majority of patients are identified at advanced stages after undergoing a number of surgical procedures or diagnostic testing, including the invasive procedures. This could be overcome by understanding the mechanism and function of differently regulated metabolites. Significant variations in the metabolites present in the different samples can be analyzed and used as early biomarkers. They could also be used to analyze the specific progression and type as well as stages of cancer type making it easier for the treatment process. The main aim of this review article is to focus on rewired metabolic pathways and the associated metabolite alterations that can be used as diagnostic and therapeutic targets in lung cancer diagnosis as well as treatment strategies.

## 1. Introduction

Lung cancer (LC) remains a burden in modern societies, and it is considered as the top cause of cancer mortality globally. Lung cancers are classified into three types: non-small cell lung cancer (NSCLC), small cell lung cancer (SCLC) and carcinoids [1]. The majority of lung cancers are NSCLC, and the majority of NSCLC are adenocarcinomas. Non-small-cell lung cancer (NSCLC), which is clinically and pathologically distinct from small cell lung cancer, accounts for around 80% of lung cancer cases [2]. NSCLC is classified into three subtypes: adenocarcinoma, squamous cell carcinoma, and large cell carcinoma.

Efforts have been undertaken in recent years to define the optimum treatment plan based on cancer sub-stage, to evaluate patients who would benefit from local intervention alone and do not require additional medical therapy, and to investigate innovative therapeutic options [3]. The discovery of specific epidermal growth factor receptor (EGFR)-activating mutations has transformed NSCLC therapy. Because of their excellent performance, EGFR-tyrosine kinase inhibitors (TKIs) have become the first-line treatment for patients with EGFR mutations [4].

In the lung cancer (LC) scenario, metabolomics permits the assessment of different variations in the quantity and type of metabolites expressed in LC. Identifying these specific metabolites can assist in distinguishing the pharmacological treatment’s nature, stage, and, perhaps, response. Most LC-specific metabolomics studies aim to create cost-effective approaches with high sensitivity and specificity for early tumor diagnosis. The metabolic activity of a cell dictates its ability to survive and consequently causes disease progression. To maintain the energy and biosynthetic precursor demands of proliferation, tumour cells adopt various metabolic methods, namely a faster rate of aerobic glycolysis [5,6]. The Warburg effect, which depicts a rapid rate of glycolysis followed by lactic acid fermentation, is one of the fundamental metabolic mechanisms that define the phenotype of tumor cells [5,7,8,9]. This article aims to provide a synopsis of recent research into the discovery of novel metabolites that show promise as biomarkers for the early detection of LC, the tracking of disease progression, and the prediction of patient survival. A better understanding of these metabolite expression could also lead to formulating a better treatment strategy in the future.

## 2. Search Strategy

The studies for this review article are selected on the basis of metabolomic studies conducted in different lung cancer subtypes. Most of the data collected are filtered for the last 10 years of data on lung cancer metabolomics. The main search terms used entirely for this review includes lung cancer and metabolites. Most of the selected studies are concentrated in the human models. This review will give a brief summary on the development of different therapeutic targets based on the significant metabolites that are rewired in the lung cancer metabolic pathways. The main platforms used for searching articles were PubMed and Google Scholar.

## 3. Importance of Serum Metabolites Collected before, during, and after Chemotherapy

Anlotinib is a novel oral tyrosine kinase inhibitor that inhibits cancer cell proliferation via targeting vascular endothelial growth factor receptors 1, 2, and 3 (VEGF)**,** fibroblast growth factor receptors 1, 2, and 3 (FGFR), platelet-derived growth factor receptor-α, and c-Kit [10,11]. Only a few patients benefit from anlotinib treatment for LC. To screen its effectiveness, a proteomics and genomics study was used to identify key metabolites (appropriate biomarker) involved after anlotinib administration. Recently, this method has been widely applied to evaluating the novel biomarker for LC diagnosis [12,13]. Mo et al. reported the 14 differential metabolites of lung adenocarcinoma patients [13]. In contrast, Zhou et al. identified the metastasis biomarkers for lung adenocarcinoma, including spermatogenesis-associated protein and elongation factor 1-alpha 2 levels [14]. Pamungkas et al. found that LC-associated factors and amino acids (AA), including retinol, bisphenol A, and L-proline, might be the potential diagnostic tool for LC [15]. Ma et al. demonstrated that using cell metabolomics, the AA metabolism regulation, HIF-1, and PI3K-Akt signalling pathways were highly related to osimertinib resistance [16]. All these research studies clearly state the importance and broad application of metabolomics to track the specific changes in LC metabolites in patients after the drug’s action [17].

Surgery is anticipated to modify metabolite profiles in LC patients, and metabolomic investigations may provide a more detailed knowledge of these operation-related changes. Circulating tumour cells (CTCs) are discharged into the bloodstream from primary or metastatic cancers [18]. CTC levels have been proven to be independently predictive of patient outcomes in both NSCLC and SCLC patients. In a study, it was observed that both pre- and post-surgery showed higher levels of sphingolipids such as ceramide and sphingomyelin in LC patients compared to controls [19].

### 3.1. Amino Acids

Pan et al. observed the anlotinib-regulated amino acids (AAs) metabolism (tryptophan, threonine, glycine, serine, phenylalanine) and AA biosynthesis (valine, leucine, isoleucine) pathways, and glyoxylate and decarboxylate pathways in vivo. These pathways are essential in cancer cell metabolism and are associated with the TCA cycle [17]. Tryptophan is a crucial AA for protein synthesis and a critical regulator in cancer progression [20]. In contrast, its metabolites are metabolized via kynurenine pathways, which play a significant role in immune response regulation [21]. Its reduction prevents tumor cells from the immune response [22]. Glycine is mostly used by cancer cells for their proliferation. Other essential AA plays a significant role in glutathione (GSH) synthesis, which regulates the immune system, and maintains the homeostasis between oxidant and antioxidant enzymes [23,24,25]. According to Hu and coworkers, glycine-associated glycocholic and glycodeoxycholic acid might be potential biomarkers for anlotinib efficacy [26]. Studies have shown that in blood, citrulline and phenylalanine are the prominent features of non-small-cell LC (NSCLC) [27]. In contrast, phenylalanine hydroxylase activity is non-functional in a malignant disease state, which can convert phenylalanine to tyrosine [28,29].

### 3.2. Lipids

The chemotherapy to LC alters its metabolism. It leads to a reduction in valine and lactate, ultimately showing relieved glycolysis. Chemotherapy also shows its effectiveness and reverses the Warburg effect. Chemotherapy affects the high serum level of lipids, choline, and isobutyrate. The cisplatin treatment to A549 cells showed an elevated lipid level but a low level of AAs and niacinamide [30]. Glycoprotein is a crucial protein in maintaining protein stability [31], whereas chemotherapy disturbs the cell membrane integrity, which results in the rupture and decomposition cell membrane. This leads to an increased concentration of lipids and glycoproteins in the blood. It has also been suggested that chemotherapy increases lipolysis via increased levels of 3-hydroxybutyrate metabolite in sera [32,33].

Similarly, the study by Xu et al. observed an increased level of choline and lipids in treated serum compared to the healthy or untreated group. They suggested that chemotherapy ruptured the cell membrane and released the lipid and phosphatidylcholines, where lipid and choline are crucial components in the cell membrane [34]. The ruptured cell membrane reconstruction demands choline degradation to the principle component [35]. Simultaneously, cell membrane integrity is needed for cancer cell proliferation, which needs principal components and phosphatidylcholines. These can be formed from choline by the action of choline kinase [36,37]. Cancer cell inhibitors inhibit cancer cell proliferation and, in most cases, decrease the principle component level and choline [38].

### 3.3. Proteins

AAs are an essential source of protein expression. Mostly the energy needed is provided from glycolysis, whereas the AAs are generated from gluconeogenesis or degradation of nutrients [34]. Chemotherapy is essential for several biological processes. It disturbs LC serum metabolites and multiple metabolic pathways, including energy metabolism alteration and biosynthesis of phosphatidylcholine and also influences microtubule function and DNA replication. So, it is possible that any chemo-drug can disturb metabolic pathways and produces metabolites—an end product [39,40]. GSH can perform cisplatinum detoxification and can repair DNA adduct [41,42]. The expression of transporter proteins is ATP7B and ABCG2 correlated with platinum resistance. However, it is suggested that Ribonucleotidereductase M1 and M2, mitogen-activated protein kinases (MAPKs), and human equilibrative nucleoside transporter 1 (hENT-1) are the group of proteins resistant to gemcitabine, taxanes or vinerolbine [43].

## 4. Types of Samples Used for Metabolite Analysis in LC

Cells, fluids, or tissues can all be used as samples for metabolomics analysis; however, biofluids such as urine, whole blood, plasma, serum, and saliva are the most frequently utilized as they can be easily acquired [44].

### 4.1. Serum/Plasma Metabolites

Molecular biomarkers in the serum metabolome could support early detection of LC in low-dose computed tomography screening programs [45]. There have been a few reports of targeted metabolic profiling of blood plasma or serum for the identification of LC biomarkers. By using liquid chromatography/mass spectrometry (LC/MS), Maeda and colleagues reported the differences in plasma amino acid profiling between healthy controls and non-small-cell LC (NSCLC) patients [46]. According to serum-based metabolomics studies conducted in recent years, serum metabolites can be used to differentiate LC types and predict severity. Thus, the approach has extraordinary potential to help diagnose LC early and manage it clinically [47,48,49,50]. Most of the metabolomics studies were conducted on Chinese, European and Korean populations whereas only a few studies were conducted on Indian populations despite the increasing prevalence of LC in the country [51]. Yu et al. found that a combination of lipid markers such as (ePE(40:4), C(18:2)CE, LPE(18:1) and SM(22:0)) can be used for the early detection of NSCLC, and in all stages of NSCLC, an increase in the level of plasma β-hydroxybutyric acid can be seen [52,53,54,55]. Earlier studies have reported lower plasma citrate levels in LC patients [56].

### 4.2. Bronchial Fluid

Due to its late identification, LC is one of the most lethal malignant neoplasms; whereas, by finding molecular markers in samples by conducting a normal bronchoscopy, and several liquid-based cytology methods, such as bronchoalveolar lavage fluid (BALF), the effectiveness for the diagnosis of LC can be improved [57]. BALF is a promising noninvasive source of protein biomarkers with good diagnostic specificity for LC, but the amount of research using BALF for markers needs to be increased [58]. The clinical diagnosis of LC has steadily incorporated BALF in recent years. According to the pertinent medical studies, tumor markers in the patient’s body’s bronchial lavage fluid had characteristics such as earlier appearance and higher concentrations compared with tumor markers in serum, making them more useful for early identification of LC [59,60]. With the help of BALF analysis, adverse effects linked to various treatment in the lungs can be detected and BALF cell-free DNA (cfDNA) testing, whose potential clinical uses are to test for LC and mutations, is found to be more precise for the detection of mutations linked to LC [57,61,62].

By using a polymerase chain reaction (PCR) method, it is possible to find functional EGFR mutations using cfDNA obtained from the BALF supernatant. According to the study by Kim et al., the expression of tumor-suppressor genes can be decreased by abnormal DNA methylation, which promotes the growth of cancer cells. For the early identification of NSCLC in BALF H-cadherin, RASSF1A, p16 and RAR beta gene methylation may be useful indicators, whereas utilizing DNA analysis and morphology at the same time is likely to be a quick and accurate technique for the identification of NSCLC [63,64]. Improved diagnostic value was demonstrated by DNA methylation biomarkers such as TERT, p16, RASSF1 and WT1 in the evaluation of cytological bronchial washings and it could be used for the analysis of DNA methylation of 7CpGs (PRR15, HOXA1, PHF11, TBX15, PDGFRA and TFAP2A genes) to detect NSCLC [65,66].

A cohort study analyzed N-proteoglycan levels in BALF from patients with LC and benign lung diseases using solidphase N-proteoglycan extraction, liquid chromatography/tandem mass spectrometry and iTRAQ labeling. Compared to benign BALF, eight glycoproteins (integrin alpha-M, lysosome-associated membrane protein 2, cullin-4B, neutrophil elastase, cathepsin D, napsin A, BPI fold-containing family B member 2, and neutrophil gelatinase-associated lipocalin) showed a two-fold increase in cancer BALF [66]. Amino acids, nucleic acids, lipids, and sugars are examples of metabolites of tumor cells that may represent cellular metabolic processes that promote the growth and development of tumors. Leukotriene B4 and cysteinyl leukotriene levels, which are byproducts of the metabolism of arachidonic acid, were shown to be significantly higher in the BALF of NSCLC patients in an early study [67].

Another study discovered that the purinergic receptors P2Y1, P2X4, and P2X7 were more highly expressed in patients with NSCLC than in those with COPD, and that this difference was the reason why the blood levels of ATP and ADP were significantly lower in NSCLC patients than in COPD patients [68]. When comparing LC patients’ BALF to healthy individuals, Callejón-Leblic et al. in his study found 42 altered metabolites and found that the levels of adenine, carnitine, choline, phosphoric acid and glycerol can be used to discriminate LC and the controls, according to ROC curve analysis, should be taken into consideration as prospective biomarkers [69]. In terms of LC diagnosis, pathology type, and other factors, the BALF and serum tumor markers detection exhibited good clinical value and relevance.

## 5. Importance of Bronchoalveolar Lavage Fluid (BALF) in Detecting Metabolites in LC Patients

Searching for early diagnostic biomarkers is extremely difficult because LC is one of the ten leading causes of mortality worldwide. Prior metabolomic investigations have not been conducted with bronchoalveolar lavage fluid (BALF) samples from patients with LC. So, researchers have been analyzing this fluid for potential new roles in LC’s metabolic process. With such a high mortality rate and a direct correlation between an early diagnosis and the success of subsequent therapy, finding a way to detect LC in its earliest stages is a formidable challenge. As a result of its proximity to the lung tissue, BALF provides a more accurate assessment of lung health than studies of peripheral biofluids such as blood or urine. Therefore, this work is a novel addition to the literature on the subject, expanding our knowledge of LC’s pathophysiology and the potential applications of effective biomarkers. By instilling a liquid into one or more lung segments and then aspirating it, the bronchoalveolar lavage fluid (BALF) can be collected throughout exploratory research of patients with lung illnesses [70]. Due to its proximity to the lung tissue, BALF provides a more accurate assessment of the health of the lungs.

Beyond cytology, few researches have looked at the significance of BALF in detecting lung cancer. BALF cfDNA may be used as a liquid biopsy medium that helps in discriminating small malignant tumours from benign tumours [71]. Another study using BALF found that TIMP-1, Lipocalin-2, and Cystatin-C plasma levels were observed to be considerably higher in adenocarcinomas and squamous cell carcinomas when compared to controls [72].

### 5.1. Its Role in Understanding the Molecular Characteristic by Free DNA and Exosomes Analysis in LC

Cell-free DNA (cfDNA) is found in the blood that has been released into the bloodstream due to apoptosis or necrosis of cells and can be isolated from plasma [73]. The cfDNA molecules in the bloodstream are already eliminated within an hour of release. Research has shown that the amount of cfDNA from healthy cells in the plasma of healthy people is relatively low (only 10–15 ng/mL). Conversely, cfDNA levels rise in response to tissue stress, such as during physical exertion, inflammation, surgery, or tissue injury [74]. Cell-free DNA (cfDNA) is a subset of ctDNA that encodes the genes of tumor cells and is liberated by apoptotic or necrotic tumor cells [75]. Double-stranded ctDNA fragments typically range in size from 160 to 200 base pairs [76], which is about the same as the average size of a nucleosome. The percentage of ctDNA can range widely, from <0.1% to >90% [77,78]. Currently, the primary methods for detecting ctDNA are the amplification refractory mutation system (ARMS), digital PCR, and next-generation sequencing (NGS). All these methods can analyze the quality and quantity of ctDNA and accurately find ctDNA through DNA amplification [79].

It was not until the 1980s that reticulocytes were discovered to be the first cells to release exosomes, which are membrane-bound vesicles [80]. They are classified as EVs (extracellular vesicles) and range in size from 40 to 160 nm (average diameter of about 100 nm). They are secreted from the endosome system and are produced by a wide variety of cell types. Multivesicular bodies form due to the persistent invagination of the plasma membrane; the subsequent fusion of these entities with the plasma membrane and secretion occurs due to various mechanisms working in concert. Multivesicular bodies’ ability to communicate with other cellular vesicles and organelles enriches the exosome’s component pool [81].

Based on their size, shape, and place of origin, EVs are classified as either exosomes, oncosomes, microbubbles, or apoptotic bodies. Saliva, blood, bronchoalveolar lavage (BAL) fluid, sputum, and other bodily fluids contain exosomes [82]. Cell membrane invaginations lead to the formation of endosomes, which in turn give rise to multivesicular bodies. These entities are then secreted from the cell and acquire a diameter of 30–150 nm as exosomes. Proteins, lipids, nucleic acids (DNA and RNA), and other cellular data are transported via exosomes [83].

### 5.2. Understanding the Immune Reaction by Analysis of Immune Cells and Mediators

Immune checkpoint inhibitors (ICIs) are novel immune-system-based therapies demonstrated to increase survival in advanced NSCLC. Unlike conventional chemotherapeutic medicines, ICIs enhance the reaction of the body to tumors. This distinct method of action has led to the identification of class-specific adverse effects. These toxicities, labeled immune-related adverse events, can impact many organ systems, including the lungs. Immune-mediated lung injury due to ICI usage, also referred to as checkpoint inhibitor pneumonitis (CIP), was found to occur in approximately 3–5% of patients that received ICIs. Although, the incidence of this condition in the real world may be higher now that ICIs are utilized in nonclinical trial settings [84]. Immunotherapy using ICIs is occasionally exacerbated by noninfectious inflammation of various organs related to autoimmunity, termed immune-related adverse events (irAEs). This inflammation affects the lungs often, and the term checkpoint inhibitor pneumonitis (CIP) was coined [84]. The treatment of choice is glucocorticosteroids and other immunosuppressive drugs. BAL appears to be an ideal diagnostic material for CIP [85].

## 6. Major Metabolic Pathway Changes Involved in Lung Caner

Various metabolic changes occur during cancer progressions. The altered downregulation and upregulation of different metabolites that differed from the normal conditions of cell are understood as metabolic reprogramming [86]. Understanding these metabolic alterations and identifying the significant metabolites different from the normal metabolites will help in introducing and testing novel treatment strategies and targeted therapies for LC patients. To confirm metabolomic pathway alterations, quantitative proteomics analysis of the major enzymes found in the disrupted pathways was utilized and a total of 13 unique biomarkers associated to metabolic disruption of NSCLC morbidity were found, which were implicated in four key pathways, including glycine, serine, and threonine metabolism, aminoacyl-tRNA biosynthesis, tyrosine metabolism, and sphingolipid metabolism. The proteomics study revealed a clear variation in the expression of important enzymes in these pathways, such as 3-phosphoglycerate dehydrogenase, phosphoserine phosphatase, tyrosinase, and argininosuccinic acid catenase [87]. Several metabolomics studies have gathered important information over the past two decades that will help us better understand LC and also led to the discovery of novel biomarkers [88].

### 6.1. Glycolysis

Otto Heinrich Warburg, in the 1920s, explained cancer metabolism, where he postulated that cancerous cells take up the glucose and in turn release lactate in elevated levels when compared to normal cells. Cancer is now considered as a metabolic disease due to various changes occurring in the metabolic pathways, including glucose metabolism. The metabolism of glucose varies between and within human tumours [5]. A recent study, conducted in 2016, showed the difference in glycolysis occurring between normal and LC patients [89].

Elevated levels of glucose transporters (GLUTs) were found to be involved in LC metabolism when compared to healthy cells [90]. GLUTs are involved in the circulation of glucose molecules in blood. Preclinical studies have shown that among all the reported GLUT isoforms, GLUT3 and GLUT5 were found to be higher [91]. This suggests that the metabolic changes also increase due to the higher glucose intake in the presence of elevated GLUTs. Increased levels of GLUT1 were also reported in other types of cancer including breast, brain, ovarian and pancreatic.

#### The Warburg Effect

During the process of glycolysis, glucose is broken down into pyruvate resulting in production of 2 NADH and ATP molecules. In aerobic conditions, this pyruvate is further oxidized into CO2 by TCA cycles and OXPHOS (oxidative phosphorylation). The combination of these pathways resulted in the production of 32 ATP molecules which started with one glucose molecule, while during anaerobic conditions, pyruvate is converted to lactate and secreted in the form of monocarboxylate transporters (MCT). In lung tumor cells, it was observed that glucose is metabolized via lactic acid fermentation even during aerobic conditions, which is referred to as the Warburg effect [92]. This finding laid the groundwork for current tumor identification and surveillance using 18-fluoro-2-deoxyglucose positron-emission (FDG-PET) and an insight into the metabolism of cancer [93].

The Warburg effect points out that cancer cells mostly use glycolysis and not OXPHOS for the production of energy. Tumor M2-Pyruvate Kinase (PKM2) is a glycolytic enzyme that is required for the cancerous transformation and is found to be elevated in LC and several other cancers [94,95]. Although evidence points out the involvement of glycolysis rather than OXPHOS in cancer cells, some research findings identified that OXPHOS produced the majority of the ATPs in the cancer cells [96]. It was also observed that mitochondrial OXPHOS and glycolysis was significantly increased in non-small-cell lung tumors (NSCLCs)[97]. Both cancer cells and carcinoma-associated fibroblasts (CAFs) create lactate by glycolysis, which is then converted to pyruvate and reaches the mitochondria of aerobic LC cells, where it undergoes OXPHOS to make ATP. One significant method by which cancer tissue maintains the equilibrium of the interactions between glycolytic and oxidative cells is through this lactate shuttle, primarily through MCT1 and MCT4 [98]. It is well known that MCT1 is the entrance of lactate into tumor cells whereas MCT4 carries lactate out of the cell [32,99].

### 6.2. Amino Acid

#### 6.2.1. Glutamine: Glutaminolysis

According to the Warburg effect, cancer cells depend on glycolysis and pyruvate is converted to lactate. This limits the uptake of pyruvate for the TCA cycle. Along with this, the TCA cycle metabolites are redirected during the anabolic process occurring in the proliferating cancer cells. In order to compensate for these reduced intermediate products occurring in the TCA cycle, LC cells increase the uptake of glutamine [100,101]. When compared to other cancer types, there is a significant increase in the level of glutamine expression in LC tissue NSCLC. Once glutamine enters the cell, it is converted into glutamate which is further converted to α ketoglutarate with the help of the enzyme called glutaminase. This process is called glutaminolysis [102]. Glutaminase is expressed in mammals in two distinct isoforms: kidney-type (GLS1) and liver-type or GLS2 [103]. The GAC variant which is smaller splice variant of the GLS1 is most commonly upregulated in LC.

##### Glutaminolysis and Associated Transporters

**Figure 1:**
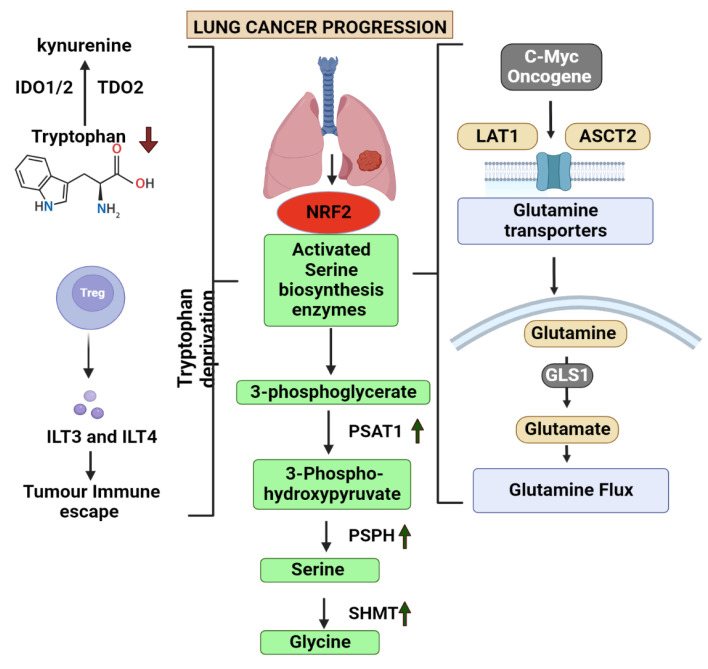
This figure explains the role of 4 main amino acid alterations involved in lung cancer. It also depicts the common degregulated pathway involved in the metabolism of amino acids glutamine, tryptophan, serine and glycine and the associated metabolites. (IDO1/2—Indoleamine-2,3-dioxygenase, TDO2—Tryptophan 2,3-Dioxygenase, ILT3/4—Immunoglobulin-like transcript 3/4, NRF2—Nuclear factor erythroid 2-related factor 2, PSAT1—phosphoserine aminotransferase 1, PSPH—Phosphoserine phosphatase, SHMT—Serine hydroxymethyltransferase, GLS1—Glutaminase1, LAT1—L-Type Amino Acid Transporter 1, ASCT2—ASC amino acid transporter 2. Arrow up—Upregulated, Arrow down—Deregulated).

The uptake of these glutamine into the cancer cells are mediated by alanine-serine-cysteine-transporter-2 (ASCT2 or SLC1A5) and it was observed that there was increased expression of these transported in LC patients [104,105]. L-type amino acid transporter 1 (LAT1) or SLC7A5/SLC3A2 are another set of molecules associated with the glutamine addiction (Figure 1). An increased expression of these molecules were observed in the LC cells [106]. The LAT1 permits the exchange of glutamine for essential amino acids such valine, methionine and phenylalanine. C-MYC is highly expressed in LC cells, which causes glutamine addiction and the production of nearly all glycolytic enzymes [107].

According to previous studies, oncogene activation of signalling pathways and transcription factors including MYC results in metabolic alterations that are sufficient to meet the elevated metabolic needs of cancer cell growth [7]. Non-targeted metabolomics analysis of intermediates from the glutamine pathway in tissue, serum, urine, and bronchoalveolar lavage fluid from LC patients and healthy individuals revealed substantial differences that were linked to the development of LC [108]. SLC38A3 is another transporter that regulates the PDK1/AKT signalling cascade and facilitates the metastasis of NSCLC by controlling the transport of glutamine as well as histidine. This points out that it has a clear clinical significance and potential for the treatment of NSCLC [109]. HIF-2α regulates and upregulates the expression of SLC1A5, whilst HIF-1α activates SLC38A1 and offers many ways to modify the glutamine level in the cell [110,111].

#### 6.2.2. Serine and One-Carbon Metabolism

In order to sustain their energy supply, cancer cells often employ glycolysis, and serine synthesis pathway is a crucial component of this process. Serine biosynthesis is fueled by the precursor, 3-phosphoglycerate and glutamate, which are produced by the glycolysis and glutaminolysis pathways, respectively [112]. Serine that is obtained during the glycolysis process can be converted into glycine which then act as a one-carbon source for the one-carbon metabolism [113]. The precursor 3-phosphoglycerate is oxidized and catalysed into 3-phosphoserine and α-KG with the help of an enzyme called phosphoserine aminotransferase (PSAT1) which is the converted to serine with the help of another enzyme called 1-3-phosphosrine phosphatase [114]. Studies has provided evidences that PSAT1 is elevated in NSCLC. PSAT1 overexpression impairs the prognosis of cancer by promoting cancer cell proliferation, metastasis, as well as chemoresistance discovered that NRF2 regulates the expression of the important serine synthesis enzymes PHGDH, PSAT1, and SHMT2 through the activation of ATF4 to promote glutathione and nucleotide formation [115]. In response to serine deprivation, ATF4 stimulates serine biosynthesis genes in NSCLC cells [116]. The methionine and folate cycles are part of one carbon metabolism, which is crucial for maintaining genomic stability and regulating nucleotide metabolism. The role of enzymes involved in this metabolism is crucial in the progression of cancer cells. Methylenetetrahydrofolate Dehydrogenase 1 and 2 (MTHFD2/1), an enzyme in the one-carbon metabolism was found to be associated with poor prognosis in LC patients [117].

#### 6.2.3. Glycine Metabolism

The progression and growth of cancer has been found to depend on glycine metabolism. Lung-tumour-initiating cells had elevated levels of glycine decarboxylase, which is the enzyme responsible in the glycine cleavage pathway. In NSCLC, glycine decarboxylase is essential for tumour-initiating cells. The accumulation of glycine is transformed into harmful metabolites including methylglyoxal and aminoacetone when glycine decarboxylase is blocked in cells that have high serine hydroxy-methyltransferase 2 (SHMT2) levels, which causes cell growth arrest [118].

#### 6.2.4. Tryptophan Metabolism

Tryptophan is crucial for forming the cytoskeleton and biosynthesizing of cellular proteins, just as with other essential amino acids. About 10% to 20% of tryptophan in the blood is free amino acid which degrades via kynurenine (KYN) pathway while the majority of it adheres to albumin [119]. Recent research has identified that the plasma tryptophan and its metabolites in the KYN pathways was decreases in NSCLC patients while 3-hydroxyanthranilic (3-HAA) 3-HAA levels were increased [120]. The main enzymes, indoleamine-pyrrole 2,3-dioxygenase (IDO)1, IDO2, and tryptophan 2,3-dioxygenase (TDO) are the ones that are responsible for the KYN pathway activities, where the tryptophan is first converted to formyl-kynurenine which is quickly reduced to KYN [121]. It was noted in a study that the level of IDO1 was elevated in the LC patient samples and the ratio between the YN and tryptophan was also greater in these cases [122].

### 6.3. Fatty Acid Synthesis Pathway5

Due to the enhanced activities of glucose, ATP production and several carbon-associated intermediates are readily available for the biosynthesis of various lipids [123]. FASN is which is an enzyme required for the de novo biosynthesis of the fatty acids, is found to be expressed in high levels in NSCLC patients [124]. FASN was also found to be involved in the metabolism of glucose by downregulating the AKT/ERK pathway which led to changes in the LC cells [125]. ATP citrate lyase enzyme (ACLY) is another enzyme which was found to be upregulated in the lung adenocarcinoma cancers which was associated with poor prognosis [126]. AcCoA carboxylase (ACC1/2) was also found in significantly higher levels in NSCLC patients when compared to healthy samples [127].

B7-H3, which is a glycoprotein, was found to be upregulated. B7-H3 is involved in the control of FASN enzyme in the fatty acid synthesis process and thus also modulates the functioning of SREBP-1 in the LC patients [128]. Further studies are required to explore more metabolites involved in the fatty acid synthesis occurring in the LC cells. Fatty acid binding protein 5 (FABP5) is involved in the regulation of the metabolism of lipids and also diverts the fatty acids to lipid synthesis. They also regulate the de novo synthesis of fatty acids which in turn modulates the functional expression of FASN and SCD1. In lung adenocarcinomas, a high level of FABP5 was found, which was related to the poor prognosis of this cancer type [129]. NFY (Nuclear factor Y) is a transcriptional factor that may identify CCAAT boxes in the promoter and is also involved in the lipogenesis process and other biological functions. NFY was also found to be upregulated in the LC samples with a poor clinical outcome [130].

#### 6.3.1. Phospholipid Metabolites

Studies have focused on the importance of fatty acids and phospholipids in NSCLC [131]. Serum lipidome screening in NSCLC patients has identified phosphatidylcholine and lysophosphatidylcholines as major diagnostic biomarkers for the LC [131]. Choline kinase, that is involved in the phosphatidylcholine synthesis was found to be over expressed in the LC patient samples [132,133]. Phosphatidylethanolamine methyltransferase (PEMT), which is an enzyme involved in the conversion process of phosphatidylcholine, was found to be increased in NSCLC samples and was consistent with a poor outcome [134].

#### 6.3.2. Sphingolipid Metabolism

Sphingolipid metabolism is found to one of the most deregulated pathways in LC progression. Ceramide is largely considered as the centre of this metabolic pathway since it connects the metabolism of several sphingolipids. Numerous studies have found elevated ceramide levels in emphysema patients who smoke and are found to be at risk of developing LC [135]. Ceramide synthase was elevated in NSCLC tissues which was done in a gene expression analysis. Sphingosine kinase 2 (SPHK2), an enzyme involved in the sphingolipid metabolism that synthesis sphingosine-1-phosphate (S1P) by phosphorylating sphingosine, has also been linked to poor survival in NSCLC (Table 1) [136].

#### 6.3.3. Increased Cholesterol Synthesis

About 80% of total cholesterol is typically produced through biosynthesis processes, and 20% comes from food. According to a new study, LC risk is increased by abnormally high or even low blood cholesterol levels [137]. In experiments conducted in vitro, it was found that the presence of 25-Hydroxycholesterol increased LC cell growth and invasion [138]. Thyroid transcription factor 1 (TTF-1) is a critical regulator of lung development and morphogenesis and directly targets ATP-binding cassette transporter A1 (ABCA1) in the LC cells. TTF-1 was found to be upregulated in the LC samples and could be used as a diagnostic marker for LC [139].

**Table 1:** Expression patterns and functions of fatty acid metabolites involved in the different types of lung cancer and its progression.

Fatty Acid Metabolite	Function	Expression Pattern	Pathway Affected	Lung Cancer Type	References
FASN	De novo biosynthesis of the fatty acids	Overexpressed	AKT/ERK pathway	NSCLC	[124]
ACLY	Degradation is blocked and leads to accumulation which further leads to increased fatty acid metabolism	Upregulated	AKT signalling pathway	lung adenocarcinoma	[140,141]
ACC1/2	Required for the de novo fatty acid synthesis that helps in growth and viability of tumours	Upregulated	-	NSCLC	[142]
B7-H3	Deregulated the mRNA expression levels of SREBP-1 and FASN	Upregulated	SREBP-1/FASN signalling pathway	lung cancer A549 and H446 cell lines.	[128]
SREBP-1	Involved in the synthesis as well as the uptake of cholesterol, fatty acids, and phospholipids	Overexpressed	SREBP-1/FASN signalling pathway	NSCLC	[143]
FABP5	Controls the lung cancer metastasis by regulating NK cell maturation process	Upregulated	PI3K/AKT/mTOR signalling	lung adenocarcinoma	[116,144]
NFY	Involved in the lipogenesis process	Overexpressed	-	lung squamous cell carcinomas	[130]
SCD1	Sustains rapid cancer cell proliferation and evades cell apoptosis	Upregulated	EGFR/PI3K/AKT signals	NSCLC	[145,146]
C16	Mediates cell proliferation	Overexpressed	Sphingolipid metabolic pathway	NSCLC	[109]
ABCA1	Regulates the level of intracellular cholesterol	Downregulated	Cholesterol metabolic pathway	Lung cancer	[147]
TTF-1	Role in morphogenesis of the lungs	Overexpressed	-	primary lung adenocarcinoma	[148]

## 7. Major Signalling Pathways and Its Role in LC

### 7.1. mTOR Signalling Pathway

Furthermore, it has been shown that genetic variations in mTOR complex components are closely related to cancer. Amplification of RICTOR, an mTORC2 component, was discovered to be associated with a poor prognosis and short survival in NSCLC, squamous cell lung carcinoma, and breast cancer (Table 2) [149]. A study of 51 Japanese patients with small cell LC (SCLC) found that 36% of the tumors showed mTOR pathway-related genetic alterations. It has been shown that phosphorylated mTOR aids in the advancement of SCLC [150]. Chronic mTOR inhibition and glycolysis reduction in LC led to an increase in glutaminolysis through a mechanism involving GSK3. Numerous malignancies that develop resistance to various treatments have been discovered to have greater glutaminolysis and/or higher levels of GLS. Combining therapy with mTOR inhibitors with the glutaminase inhibitor CB-839 is beneficial in overcoming therapeutic resistance to certain other focused inhibitors in these cancers [151]. More research is needed to find the importance and role of mTOR and also the associated metabolites associated in LC pathophysiology.

### 7.2. AMPK (AMP-Activated Protein Kinase) Signalling Pathway

It was observed that liver kinase B1 (LKB1), which is a recognized tumor suppressor, functioned as an upstream kinase of AMPK, which was considered as the first clue to AMPK being connected to cancer mechanisms. Studies showed that LKB1 is mutated in numerous lung and cervical cancers, indicating that AMPK may be involved in tumor suppression. Autophagic death was brought to NSCLC cells by circular RNA circHIPK3 via the activation of the AMPK cascade [152,153]. In ASP4132, treated where ASP4132 is an AMPK activator in the NSCLC cells, AMPK downstream actions such as mTORC1 inhibition, PDGFR and EGFR degradation, Akt suppression, and autophagy activation were identified. It was also observed that the expression of AMPK1 is much increased in NSCLC tumor tissues [154]. In NSCLC cells, a study performed a proteomics analysis where AMPK-interacting proteins were screened and it identified the platelet isoform of phosphofructokinase 1 (PFKP), which is a rate-limiting enzyme. Additionally, it was discovered that NSCLC patients’ highly expressed PFKP was linked to poor survival [155].

### 7.3. HIF-1α Signalling Pathway

In non-small-cell LC, it is established that transforming growth factor (TGF)-beta performs a dual role in controlling glycolysis and cell proliferation. Crosstalk between TGF and HIF-1 together encourages a rise in the expression ratio of PKM2/PKM1 which, in turn, encourages tumour cell glycolysis. It was also noted that, in contrast to PKM1, it was discovered that PKM2 was strongly expressed in LC tissues. In mice lung tumors and cancer-associated fibroblasts from human LC tissues, HIF-1 was substantially expressed [156]. More studies are required to understand the role and mechanism involved the changes in different metabolites associated with LC and HIF-1α signalling pathway.

**Table 2:** Metabolites associated with different signalling pathways involved in the progression of lung cancer.

Signalling Pathway	Metabolite	Cancer Type	Function	Expression Pattern	References
mTOR signalling pathway	S6K1	NSCLC Cancers	S6K1 is a key kinase responsible for Mxi1 phosphorylation and downregulation	Over expressed	[157]
	4E-BP1/eIF-4E	NSCLC Cancers	Promotes the translation of specific pro-oncogenic proteins that regulate cell survival, cell cycle progression, angiogenesis, energy metabolism, and metastasis	Over expressed	[158]
	RICTOR	NSCLC and SQCLC tumours	Akt hyperactivity and tumour aggravation	Over expressed	[149,159]
AMPK signalling pathway	PFKP	NSCLC	PFKP facilitated the mitochondrial recruitment of AMPK which subsequently phosphorylated ACC2 to promote long-chain fatty acid oxidation	Over expressed	[155]
	Circular RNA circHIPK3	NSCLC	Functions as an oncogene and autophagy regulator	Over expressed	[153]
HIF-1α signalling pathway	HOXB7	lung adenocarcinoma	HOXB7 upregulates several canonical SC/iPSC markers and sustains the expansion of a subpopulation of cells with SC characteristics	Over expressed	[160]
	HOTAIR	non-small cell lung cancer	HOTAIR-enhanced cancer cell proliferation, migration, and invasion under hypoxic conditions	Over expressed	[161]

## 8. Metabolite Profiles Related to Radio-Resistance

Metabolic changes can cause radio resistance, and it has been demonstrated that changes in the glycolytic pathway aid in the development of radio resistance. The effects of radiotherapy mostly depend on glucose metabolism, while mitochondrial metabolic changes can also play a role in this process [162]. Results of radiation are negatively impacted by cells that are hypoxic. In LC cells, hypoxia can promote the expression of EGFR and NRF2, leading to the development of radio resistance, as well as the activation of HIF1 expression, which controls the adaptive cellular responses to hypoxia [163]. HIF1 can boost the expression of the autophagy gene BECN1 and also c-JUN, which causes radio resistance in cells of LC [164].

## 9. Metabolomic-Based Biomarkers for Lung Cancer Smokers and Non-Smokers

Smoking is the leading cause of cancer death, accounting for about 64.2% of all LC associated deaths worldwide in 2019. In nonsmokers, secondhand smoke, as well as other environmental and genetic variables, are possible risk factors for the development of LC. Secondhand smoking is responsible for around 6% of global nonsmoker deaths from LC in 2019 [165]. Carotene, vitamin E, vitamin C, copper, zinc, and selenium supplementation was shown in experiments to significantly lower serum/plasma levels of cigarette smoking oxidative stress biomarkers in LC smokers. However, because of its link with cigarette-smoking-induced oxidative stress, iron has an oxidant impact, making it difficult to prescribe as a supplement for LC therapy in smokers [166]. A study of current smokers found a strong positive relationship between the levels of cotinine and its risk towards lung cancer. Former smokers as well as the never-smokers were found to have cotinine levels consistent with active smoking [167]. Cigarette smoking is associated with reduced incidence of EGFR mutations and serious adverse events. EGFR mutations are seen in around 10% of lung cancers in the United States, compared to 35% among East Asians. The existence of EGFR mutations is a critical predictor of EGFR-tyrosine kinase inhibitor activity [168]. More research is needed to identify the potential risks for lung cancer as well as the aspects related with a better outcome to EGFR-TKI.

## 10. Common Metabolomics Techniques Employed to Study LC Metabolites

The metabolite refers to a variety of small molecule compounds (molecular weight 1500 Da), which make the analysis of the data relatively simple as compared to proteomes and genomes. Cells and organs depend on metabolites to maintain their normal physiological function, and they are involved in intercellular communication. Metabolomics, unlike genomics, proteomics and transcriptomics, identify all the small molecule metabolites in organisms and tell of what has happened in organisms directly and accurately [169].

Traditional metabolomics had drawbacks such as small metabolome coverage and only a limited number of metabolites could be accurately identified. In contrast to it, the next-generation metabolomic technology based on high performance liquid chromatography coupled with tandem mass spectrometry (LC-MS/MS) has greatly increased both sensitivity and accuracy. Next-generation metabolomics is developing into a potent new technology for research into the chemistry and systems biology of health and illness, as well as for the detection and treatment of cancer [170]. Nuclear magnetic resonance (NMR), gas chromatography/mass spectrometry (GC/MS), and liquid chromatography/mass spectrometry (LC/MS) are common metabolomics techniques used to diagnose LC [171]. Metabolomics can shed light on how cancer develops from a metabolic standpoint [172]. Due to its high sensitivity and high throughput, LC/MS is the most frequently used technique. Researchers have used the blood metabolomes of patients with NSCLC and healthy controls using ultra-high performance liquid chromatography/quadrupole time-of-flight mass spectrometry (UPLC/Q-TOF MS) [171].

Another study, where a UPLC-MS/MS-based analytical approach was utilised for NSCLC plasma biomarkers, proved to have the ability to show the stages and development of this malignancy in a practical and reliable manner. In this study, 11 biomarkers were identified which included hormones, amino acids, glucose levels, and purines [173,174]. A non-targeted metabolomics technique using liquid chromatography/mass spectrometry was performed to investigate the metabolic response of NSCLC patients to EGFR-TKIs or PD-1/PD-L1 inhibitors and it was found that they could induce variations in the levels of amino acids, fatty acids, carnitine, and lipids [175]. There was a significant difference between the amino acid and lipid metabolism observed in the above study when compared to untreated newly diagnosed patients. Differential expression of different metabolites was also studied in small cell lung cancer (SCLC) using NMR metabolomics, where significant changes in the healthy and control samples were detected. Some of the significantly altered metabolites in this study include 3-HBA, Acetoacetic acid, glutamic acid, leucine, methionine, and LDL-6 triglyceride [176,177,178].

Metabolite analysis of blood plasma using ^1^H-NMR spectroscopy holds significant promise for early cancer detection and understanding metabolic abnormalities in cancer. Furthermore, because it only takes a few minutes to produce a spectrum, ^1^H-NMR is well suited for high-throughput screening [179,180]. However, NMR has shortcomings, the most significant of which are its inherent poor sensitivity and signal overlap. ^1^H-NMR-based metabolomics developed resulted in an OPLS-DA model that correctly classified 78% of lung cancer patients and 92% of controls. Another study using NMR metabolomics indicated that the following metabolite biomarkers, isoleucine, acetoacetate, and creatine, along with the two NMR signals of N-acetylated glycoproteins and glycerol, might possibly be effective in identifying lung cancer stages [181].

The pathways implicated in lung cancer prognosis are largely engaged in metabolism, such as tyrosine metabolism and DNA replication process. Meanwhile, cancer-related signalling is mediated by diagnosis-related pathways such as the MAPK signalling system and the GnRH signalling circuit. This suggest that the markers for LC diagnosis and prognosis appeared completely different. Therefore, understanding the difference between them using pathway analysis is important.

## 11. Platforms of Next-Generation Metabolomics and Its Importance in Precision Medicine

Using genomic data, precision medicine aims to deliver the right treatment at the right time to the right patient. As a result of next-generation sequencing technology, it is possible to sequence many genes simultaneously in a rapid and accurate manner [182]. Next-generation metabolomics plays an essential role in precision medicine as an effective supplement to systems biology and gene sequencing. Any kind of biological fluids or tissues can be used as samples for next-generation metabolomics. The development and use of high-resolution tandem time-of-flight mass spectrometry (TOF/TOF), fast scanning tandem mass spectrometry (MS/MS), and large-scale metabolites identification databases (e.g., HMDB & KEGG) have all contributed to the emergence of the next-generation of metabolomics after 2010. Data processing requirements for next-generation platforms are higher than for older platforms, which use extensive mass spectrum databases to identify compounds. Only a small fraction of the thousands of ions and peaks that are found can be correctly identified [170].

## 12. Conclusions

Identifying different metabolites from diagnostic biofluids has a significant role in understanding the biological function of a particular disease progression. In LC models, several metabolomic studies have been undertaken to focus on the differently expressed metabolites. Metabolic alterations in the tumour cells are modified owing to changes in signalling pathways, protein expression, and other molecular processes, as well as particular biochemical alterations which during the cancer progression. Previous studies have reported several deregulated pathways and their metabolites playing a role in the progression. The major metabolic pathways and the significantly altered metabolites discussed in this review will give a better outline for identifying the potential biomarkers and therapeutic targets in lung cancer.

Certain metabolites are critical in differentiating the tumour types, resulting in a better diagnosis of the LC. Observing the metabolic alterations in biofluids before, during, and after chemotherapy, could be beneficial for detecting tumour growth. Understanding of intermediates that alter uniquely in distinct types of lung tumors may not only help with clinical diagnosis, but may also identify relevant molecular targets for the design of novel anticancer treatments, thereby improving overall patient survival. Despite the identification of about 150 metabolites or more that are linked to lung cancer, there continues to be more variation due to a lack of standardized patient cohorts and testing techniques. More research investigations are required to fully understand the function and expression patterns of these metabolites which could serve as novel biomarkers for detection as well as therapeutic targets in the future.

## Data Availability

Data are available from the authors on request (A.V.G.).
