# Correction: Kannampuzha et al. A Systematic Role of Metabolomics, Metabolic Pathways, and Chemical Metabolism in Lung Cancer. *Vaccines* 2023, *11*, 381

**DOI:** 10.3390/vaccines12121421

**Published:** 2024-12-17

**Authors:** Sandra Kannampuzha, Anirban Goutam Mukherjee, Uddesh Ramesh Wanjari, Abilash Valsala Gopalakrishnan, Reshma Murali, Arunraj Namachivayam, Kaviyarasi Renu, Abhijit Dey, Balachandar Vellingiri, Harishkumar Madhyastha, Raja Ganesan

**Affiliations:** 1 Department of Biomedical Sciences, School of Biosciences and Technology, Vellore Institute of Technology (VIT), Vellore 632014, India; 2 Centre of Molecular Medicine and Diagnostics (COMManD), Department of Biochemistry, Saveetha Dental College & Hospitals, Saveetha Institute of Medical and Technical Sciences, Saveetha University, Chennai 600077, India; 3 Department of Life Sciences, Presidency University, Kolkata 700073, India; 4 Stem Cell and Regenerative Medicine/Translational Research, Department of Zoology, School of Basic Sciences, Central University of Punjab (CUPB), Bathinda 151401, India; 5 Department of Cardiovascular Physiology, Faculty of Medicine, University of Miyazaki, Miyazaki 889-1692, Japan; 6 Institute for Liver and Digestive Diseases, College of Medicine, Hallym University, Chuncheon 24252, Republic of Korea

Following the publication of paper [1], concerns were brought to the attention of the Editorial Office regarding some incorrect statements and wrong references cited. Adhering to our complaint’s procedure, an investigation was conducted by the Editorial Office and Editorial Board, which concluded that a corrigendum to the paper should be published to amend the mistakes. The authors would like to make the following corrections:

In the Introduction section, the second sentence of the first paragraph, “Lung cancers are classified into two types: non-small-cell lung cancer (NSCLC) and small cell lung cancer (SCLC)”, should be changed to, “Lung cancers are classified into three types: non-small cell lung cancer (NSCLC), small cell lung cancer (SCLC) and carcinoids [1]”, with the following references added at the end of the sentence, which was cited as reference 1 in the original paper:

The Clinical Lung Cancer Genome Project (CLCGP) and Network Genomic Medicine (NGM). A genomics-based classification of human lung tumors. *Sci. Transl. Med*. **2013**, *5*, 209ra153.

The fourth sentence, “This kind of lung cancer represents about 30% of all lung cancers and around 50% of NSCLCs”, should be removed. The original reference 1 cited at the end of the fifth sentence, “non-small-cell lung cancer (NSCLC), which is clinically and pathologically distinct from small cell lung cancer, accounts for around 80% of lung cancer cases”, should be replaced with the following one, numbered as reference 2 in the updated paper:

Zugazagoitia, J.; Molina-Pinelo, S.; Lopez-Rios, F.; Paz-Ares, L. Biological therapies in nonsmall cell lung cancer. *Eur. Respir. J*. **2017**, *49*, 1601520.

Due to the newly added reference, the change in the order of the references will be continued throughout the paper hereafter.

In the second paragraph of Section 5.1, the first sentence, “It was not until the late 1980s that reticulocytes were discovered to be the first cells to release exosomes, which are membrane-bound vesicles [79]”, should be changed to “It was not until the 1980s that reticulocytes were discovered to be the first cells to release exosomes, which are membrane-bound vesicles [80]”. The reference cited here must be paired with the following paper:

Colombo, M.; Raposo, G.; Théry, C. Biogenesis, secretion, and intercellular interactions of exosomes and other extracellular vesicles. *Ann. Rev. Cell Dev. Biol*. **2014**, *30*, 255–289.

In the third paragraph of Section 5.1, at the end of the second sentence, the following reference should be cited as 82:

Lorenc, T.; Chrzanowski, J.; Olejarz, W. Current perspectives on clinical use of exosomes as a personalized contrast media and theragnostic. *Cancers* **2020**, *12*, 3386.

In the third paragraph of Section 5.1, the sentence “These entities are then secreted from the cell and acquire a diameter of 30–100 nm as exosomes” should be changed to “These entities are then secreted from the cell and acquire a diameter of 30–150 nm as exosomes”. The original reference 82 cited in the last sentence should be re-ordered as 83 and replaced with another reference:

O’Brien, K.; Breyne, K.; Ughetto, S.; Laurent, L.C.; Breakefield, X.O. RNA delivery by extracellular vesicles in mammalian cells and its applications. *Nat. Rev. Mol. Cell Biol*. **2020**, *21*, 585–606.

In the first paragraph of Section 6.1, the following reference should be added at the end of the last but one sentence, “The metabolism of glucose varies between and within human tumours [5]”, which was already cited as reference 5 in the updated paper:

Warburg, O. On the origin of cancer cells. *Science* **1956**, *123*, 309–314.

In the third paragraph of Section 6.1, the following reference marked as 92, originally at the end of the last sentence, should be replaced with the following and will be re-ordered as reference 93:

Dooms, C.; van Baardwijk, A.; Verbeken, E.; van Suylen, R.J.; Stroobants, S.; De Ruysscher, D.; Vansteenkiste, J. Association between 18F-fluoro-2-deoxy-D-glucose uptake values and tumor vitality: prognostic value of positron emission tomography in early stage non-small cell lung cancer. *J. Thorac. Oncol*. **2009**, *4*, 822–828.

In Table 1, the reference cited as 147 in line “ABCA1” should be replaced with the following one: Wu, K.; Zou, L.; Lei, X.; Yang, X. Roles of ABCA1 in cancer. *Oncol. Lett*. **2022**, *24*, 349.

In Section 7.2, “AMPK (AMP-Activated Protein Kinase) Signalling Pathway”, after the third sentence, the following reference numbered as 153 should be added along with 152:

Chen, X.; Mao, R.; Su, W.; Yang, X.; Geng, Q.; Guo, C.; Wang, Z.; Wang, J.; Kresty, L.A.; Beer, D.G. Circular rna circhipk3 modulates autophagy via mir124-3p-stat3-prkaa/ampkα signaling in stk11 mutant lung cancer. *Autophagy* **2020**, *16*, 659–671.

The original reference 152 should be removed from the fourth sentence of Section 7.2: “In ASP4132, treated where ASP4132 is an AMPK activator in the NSCLC cells, AMPK downstream actions such as mTORC1 inhibition, PDGFR and EGFR degradation, Akt suppression, and autophagy activation were identified.” In the last sentence of Section 7.2, the reference cited should be replaced with the following one and ordered as 155:

Chen, J.; Zou, L.; Lu, G.; Grinchuk, O.; Fang, L.; Ong, D.S.T.; Taneja, R.; Ong, C.N.; Shen, H.M. PFKP alleviates glucose starvation-induced metabolic stress in lung cancer cells via AMPK-ACC2 dependent fatty acid oxidation. *Cell Dis*. **2022**, *8*, 52.

In the third sentence of Section 7.3, “HIF-1α Signalling Pathway”, the originally cited reference 154 should be removed. In the fourth sentence of the same paragraph, the originally cited reference 143 should be replaced with the following one cited as 156:

Huang, Y.; Chen, Z.; Lu, T.; Bi, G.; Li, M.; Liang, J.; Hu, Z.; Zheng, Y.; Yin, J.; Xi, J.; et al. Hif-1α switches the functionality of tgf-β signaling via changing the partners of smads to drive glucose metabolic reprogramming in non-small cell lung cancer. *J. Exp. Clin. Cancer Res. CR* **2021**, *40*, 398.

In Table 2, all the references cited should be shifted two numbers ahead, except for the original reference 148, which should be changed to reference 149 in line “RICTOR”, and the original reference 146 should be changed to reference 155 in line “PFKP”.

In Section 9, the original reference 162 cited in the third sentence should be replaced with a new reference ordered as 165: Lai, X.; Li, C.; Yang, Y.; Niu, M.; Yang, Y.; Gu, S.; Hou, W.; Chen, L.; Zhu, Y. Global estimates of rehabilitation needs and disease burden in tracheal, bronchus, and lung cancer from 1990 to 2019 and projections to 2045 based on the global burden of disease study 2019. *Front. Oncol*. **2023**, *13*, 1152209. The reference cited in the last but one sentence should be 168:

168. Zhang, P.; Nie, X.; Bie, Z.; Li, L. Impact of heavy smoking on the benefits from first-line EGFR-TKI therapy in patients with advanced lung adenocarcinoma. *Medicine* **2018**, *97*, e0006.

In the last sentence of the first paragraph in Section 10, the reference should be changed to the following one ordered as 169:

169. Wishart, D.S. Emerging applications of metabolomics in drug discovery and precision medicine. *Nat. Rev. Drug Discov*. **2016**, *15*, 473–484.

In the fourth paragraph of Section 10, the two references 177,178 cited at the end of the paragraph should be removed, and the following reference should be added there as reference 181: Song, Z.; Wang, H.; Yin, X.; Deng, P.; Jiang, W. Application of NMR metabolomics to search for human disease biomarkers in blood. *Clin. Chem. Lab. Med*. **2019**, *57*, 417–441.

In the last paragraph of Section 10, the originally cited reference 179 should be removed.

With this correction due to the missing citations, the order of references has been adjusted accordingly. The authors state that the scientific conclusions are unaffected. This correction was approved by the Academic Editor. The original publication has also been updated.
